# Pragmatic Management of Drug-Resistant Tuberculosis: A Qualitative Analysis of Human Resource Constraints in a Resource-Limited Country context—Ethiopia

**DOI:** 10.3389/ijph.2021.633917

**Published:** 2021-08-09

**Authors:** Kirubel Manyazewal Mussie, Christoph Gradmann, Solomon Abebe Yimer, Tsegahun Manyazewal

**Affiliations:** ^1^ Institute of Health and Society, University of Oslo, Oslo, Norway; ^2^ Institute for Biomedical Ethics, University of Basel, Basel, Switzerland; ^3^ Department of Microbiology, Unit for Genome Dynamics, University of Oslo, Oslo, Norway; ^4^ Coalition for Epidemic Preparedness Innovations, Oslo, Norway; ^5^ Centre for Innovative Drug Development and Therapeutic Trials for Africa (CDT-Africa), College of Health Sciences, Addis Ababa University, Addis Ababa, Ethiopia

**Keywords:** health care workers, drug-resistant tuberculosis, management of drug-resistant tuberculosis, human resource challenges, resource scarce settings, Ethiopia

## Abstract

**Objectives:** Existing evidence suggests that drug-resistant tuberculosis (DR-TB) remains a huge public health threat in high-burden TB countries such as Ethiopia. The purpose of this qualitative study was to explore the challenges of healthcare workers (HCWs) involved in providing DR-TB care in Addis Ababa, Ethiopia.

**Methods:** We conducted in-depth interviews with 18 HCWs purposively selected from 10 healthcare facilities in Addis Ababa, Ethiopia. We then transcribed the audiotaped interviews, and thematically analysed the transcripts using Braun and Clark’s reflexive thematic analysis framework.

**Results:** We identified five major themes: 1) inadequate training and provision of information on DR-TB to HCWs assigned to work in DR-TB services, 2) fear of DR-TB infection, 3) risk of contracting DR-TB, 4) a heavy workload, and 5) resource limitations.

**Conclusion:** Our findings highlight major human resource constraints that current DR-TB care policies need to foresee and accommodate. New evidence and best practices on what works in DR-TB care in such resource-limited countries are needed in order to address implementation gaps and to meet global TB strategies.

## Introduction

Drug-resistant tuberculosis (DR-TB) continues to be a major global health issue. According to the World Health Organization (WHO) 2020 global TB report, close to half a million people developed rifampicin-resistant TB (RR-TB), of which 78% had multidrug-resistant TB (MDR-TB) globally in 2019 [1]. DR-TB is a multifaceted illness with expensive treatment regimens, toxic medications, and a long duration of treatment, thereby creating a substantial burden on patients, healthcare providers, and the healthcare system [2]. Its management poses a significant economic burden [3]. Every year, nearly 20% of patients who started MDR-TB treatment die during the course of treatment, indicating that unsuccessful pragmatic management of the disease is costing many lives [4, 5]. Some resource-constrained, high TB-burden African countries initiated new policies of DR-TB to help expand the capacity of health facilities to treat patients with DR-TB, minimise delays to access care, and improve patient outcomes [6–8]; however, such initiatives placed new demands on already-burdened human resources. There are several DR-TB guidelines that may vary in quality and the topics they cover, which may therefore make it difficult for healthcare providers to select the optimal care for their patients [9]. Bringing deaths, disease, and suffering due to TB to zero demands that health policymakers and programme planners, inter alia, critically investigate existing gaps.

Ethiopia is one of the high-burden TB countries listed by the WHO; the country ranks third in Africa and is one of the 30 highest TB, TB/HIV, and multidrug-resistant (MDR) high-burden TB countries in the world [10]. The prevalence of all forms of TB is estimated at 261 per 100,000 population, leading to an annual mortality rate of 64 per 100,000 population [10]. Facilitated by co-infections like HIV/AIDS, and socioeconomic situations including poverty and inequality, TB remains a huge challenge to the country’s efforts in achieving key international health indicators. The pragmatic management of DR-TB is influenced by various factors, including poverty, crowded settings, poor nutrition, general public awareness of DR-TB, and drug toxicity [11–14]. The risk factors for DR-TB in Ethiopia include previous exposure to anti-TB treatment, exposure to a known MDR-TB case, history of using a poor or unknown quality of TB drugs, treatment in poorly performing control programmes, poor service delivery, co-morbid conditions associated with malabsorption, and HIV/AIDS [15–19].

Ethiopia follows the WHO’s End TB strategy for the management of DR-TB, with different implementation and monitoring guidelines in place to follow up on any progress. The programme emphasises the need to provide directly observed therapy (DOT) by expanding TB diagnostic and treatment services in healthcare facilities [20]. HCWs play a decisive role in taking these efforts further. Any HCW treating a patient for TB is assuming an important public health responsibility. To help effectively deploy this task, HCWs need to have the required capacity and working environment, while there could be different factors that affect day-to-day healthcare services and the quality of health outcomes [21–25]. Healthcare providers working with TB often face challenges, such as the risk of infection and workload [10, 26], which are also experienced by health professionals working with other global pandemics such as COVID-19 [27, 28] and HIV/AIDS [29]. HCWs play a vital role, not merely in the betterment of healthcare services but also in developing and achieving global health outcomes. Therefore, the pragmatic management of DR-TB needs to give proper attention to challenges and constraints that would hamper the day-to-day activities of DR-TB HCWs. Little is done in Ethiopia to learn from DR-TB care providers on gaps that should be addressed to ensure the effective pragmatic management of DR-TB at public healthcare facilities. The existing literature on DR-TB in Ethiopia either focuses on DR-TB patients only [30] or is a quantitative analysis of health workers’ knowledge and practices [31]. Thus, the purpose of this qualitative study was to explore the challenges of HCWs involved in providing DR-TB care in Addis Ababa, Ethiopia.

## Methods

We used a qualitative study design with inductive content analysis to systematically understand the participants’ lived perceptions and experiences of the phenomenon of interest and give meaning to the data. We conducted the study in Ethiopia—a country structurally divided into 11 autonomous administrative divisions. Addis Ababa, the capital, is among these administrative divisions, and it is the largest city in Ethiopia with the status of both a city and a state. The city has 10 sub-city administrations, with 10–15 suburbs (“Woreda” in the local language, which is equivalent to a district) in each administrative division. As the city is crowded with population and housing, there is a high risk of TB transmission, and for this reason, we chose it as the study setting.

We purposively selected a total of 18 HCWs from 10 government healthcare facilities that provide TB care and treatment services in the city. Of these, eight were primary healthcare facilities and two were TB specialized hospitals. Further information regarding participants’ distribution over these facilities is provided in Table 1, with the number of participants determined by the level of data saturation [32, 33]. Medical directors at each healthcare centre facilitated the selection of the appropriate study participants. All participants were approached for enrolment in the study and to volunteer to take part in the study by signing an informed consent form.

**TABLE 1:** Demographic characteristics of study participants. Pragmatic management of drug-resistant tuberculosis: A qualitative analysis of human resource constraints in a resource-limited country context—Ethiopia; 2017.

Name (anonymised)	Sex	Profession/title	Healthcare facilities (anonymised: Primary healthcare facilities as PHCF and TB specialised hospitals as TB-SH)	Service period as HCW
Kotno	Female	Clinical nurse	PHCF 1	1 year
Hansir	Female	Clinical nurse	PHCF 2	1 year
Kofi	Female	Laboratory technologist	PHCF 3	2.5 years
Kasge	Male	Health officer	PHCF 4	9 years
Lidmu	Male	Health officer	PHCF 5	16 years
Melsa	Male	Clinical nurse	PHCF 6	4 years
Teman	Male	Health officer	PHCF 7	9 years
Kash	Female	Clinical nurse	PHCF 8	3 months
Petol	Male	Clinical nurse	TB-SH 2	34 years
Pesar	Female	Clinical nurse	TB-SH 2	1 year
Mapet	Male	Clinical nurse	TB-SH 2	1 year
Pelas	Male	Clinical nurse	TB-SH 2	1 year
Shalpet	Female	Clinical nurse	TB-SH 2	1 year
Algon	Female	Health officer	TB-SH 1	5 years
Alto	Male	Clinical nurse	TB-SH 1	1 year
Treal	Male	Clinical nurse	TB-SH 1	2 years
Almet	Male	Laboratory technologist	TB-SH 1	5 years
Ambal	Male	Clinical nurse	TB-SH 1	2 years

Data were collected using in-depth interviews (IDIs), which were conducted between August and October 2017. The IDIs were held in a private room during working hours. The choice of time and venue for interviews was made without interference by the interviewer, while the study subjects themselves decided when and where the interviews were to be conducted. Amharic, the official language in Ethiopia, was the medium of communication used during the interviews. Three of the authors (KM, SY, and TM) were native speakers of this language. According to the primary investigator (KM), none of the study participants had any difficulties in speaking or listening in Amharic. The methodology of this study was designed to provide a comprehensive description of concepts through interactive and open discussions with the participants of the study. All the participants were interviewed by the first author (KM), with each interview lasting 45–55 min. Besides taking some notes that were later transcribed, the IDIs were audiotaped after securing the participant’s consent. Among all the study participants, only one HCW declined to be audiotaped. Moreover, notes were also taken during the interview with this participant.

The audio recordings were transcribed verbatim and reviewed against the transcripts by the PI (KM). The data analysis was commenced during fieldwork. The purpose of doing this was to identify information gaps early and to help to produce a broader description of topics throughout the interviews. This helped us to constantly modify the interview guide and hence attain a deeper understanding of the research question. Braun and Clark’s reflexive thematic analysis framework was applied as the data analysis framework. The analysis process followed six phases included in this framework: familiarization, initial coding, theme construction, reviewing themes, defining themes, and producing the report [34]. MS Word was used to manually code the data line-by-line using the interview language. This generated a total of 95 codes: words and abbreviations used to characterize ideas in the material. The number of codes produced helped to break down the data into meaningful segments without reducing them. In order to find any similarities among the codes, the authors repeatedly revised the entire document, from which they then developed broad themes that kept the data’s context. All the authors confirmed, and agreed upon, data saturation when the following key themes emerged: inadequate training to HCWs on DR-TB, the risk and fear of DR-TB infection, a heavy workload, and resource scarcity. These themes differed from one another in terms of scope; some of them were broader, while others were narrower and fell under the broad themes. A combination of both the English and Amharic languages was applied for the analysis, thereby indicating that the entire transcription was not translated into English. At the early stage of writing, English was used to write the findings, the themes, and their description, whereas quotes taken from the data to support the themes remained in the original language, Amharic. Amharic quotes were then translated into English when the write-up of the results was finished.

Ethical approval was obtained from the Norwegian Centre for Research Data, the Addis Ababa City Administration Health Bureau, and the 2 TB specialized hospitals in Addis Ababa, Ethiopia. Written informed consent was secured from all the participants before the start of the study. Fictitious names instead of real names of participants were used to guarantee the participants’ anonymity and confidentiality. Safe storage platforms were used to keep the audio files. The primary investigator (KM) was the only person who had access to the audio files, which were deleted at the end of the study.

## Results

The sample was comprised of 18 HCWs (seven women and 11 men), who at the time of the data collection were working in the selected healthcare facilities in Addis Ababa, Ethiopia. In total, 10 of them (55.5%) were from federal level TB-specialised referral hospitals that have TB research centres. The remaining eight (44.5%) were from sub-city level primary healthcare facilities. The demographic information is summarized in Table 1, with the data analysis resulting in five themes.

### Theme 1: Inadequate Training and Provision of Information on DR-TB to HCWs Assigned to Work in DR-TB Services

All of the participants were faced with new and complex knowledge when they started working with DR-TB patients. This was due to the fact that they had little or no prior knowledge about DR-TB. One participant said: “You do not get knowledge about DR-TB from school or universities. It is the work that teaches you. You learn from patients, for example, when they complain about drug side-effects and other things” (Lidmu, health officer)*.* None of the participants reported that they gained any basic knowledge about DR-TB through formal education (university studies) that actually helped them to carry out their daily tasks as DR-TB HCWs. Instead, what they primarily learned came through their work experience. Even though the participants emphasized work experience as the most important source of knowledge on DR-TB, they also reported that support from other colleagues was another important source of knowledge. One participant explained, “There are colleagues who know about DR-TB better than me. I just call and ask them anytime if I have questions” (Melsa, clinical nurse). Thirdly, the participants mentioned that the TB-specific training—which is given to HCWs who newly start working in TB treatment—gave them a brief awareness about DR-TB. In total, eight of the 18 HCWs (44.4%) received on-the-job general training about DR-TB. For the most part, every HCW who is assigned or employed to work in TB (including DR-TB) care takes a TB training that lasts for a maximum of 5 days. However, as most of our participants noted, the coverage and quality of this training were inadequate: “When we work on MDR-TB we take a five-day training only, so we do not know enough. For example, we do not know about the drugs and also about how to give good counselling to MDR-TB patients” (Algon, clinical nurse). Another participant added: “I remember taking a training. But that was just a training and I did not get good knowledge until I spent some time working with the patients (sic)” (Petol, clinical nurse).” Lastly, the participants reported that they increased their knowledge of DR-TB through the media and other resources, such as books. They read about DR-TB on the internet, books, and social media such as Facebook: “The other way I get information about DR-TB is by reading books. In addition, our hospital has its own Facebook page. I go there and read some relevant posts” (Mapet, clinical nurse).

### Theme 2: Fear of DR-TB Infection

The participants highlighted that working in DR-TB care induces fear. Using words such as “fear”, “scare”, “worry”, “frustration”, and “shock”, they expressed how working with DR-TB cases terrifies them. They become worried imagining the shock that DR-TB patients might experience when they hear that they are diagnosed with the disease. Furthermore, this worry is shared between family members of the HCWs, thus intensifying the anxiety among the HCWs. One participant said.

*Every one of us here is tense and gets frustrated when we diagnose patients with DR-TB. I was very shocked when I came across my first MDR-TB case in the lab. I felt terrified, as if I was the one diagnosed with the disease. Whenever I cough and feel sick, both me and my family become worried that I might have been infected with DR-TB. This disease is so scary that even wearing masks does not push our fear away. (Kofi, laboratory technologist)*

In addition to the fear of infection, participants noted that attending to DR-TB patients’ experiences contributes to an increased level of anxiety. For example, looking at the number of pills and injections patients take is distressing, with one HCW saying: “Looking at the number of pills they take every day, the injections, etc. makes me distressed” (Pesar, clinical nurse). We have also inquired if there were any changes in our participants’ fear as a result of spending more time at work. Those with more work experience in the health sector reported less fear than those with fewer years of work experience. One HCW who had 16 years of work experience said: “When I was assigned here (DR-TB ward), I felt so bad. I was continuously asking myself what wrong I did that I am being punished in this way. But later on, the fear started to fade away” (Lidmu, health officer)*.* Another HCW with 34 years of experience added.

*There were times when I used to hold my breath every time I met patients. This was to not inhale the TB bacteria. But this fear has reduced over time. I see my colleagues walking around without putting on masks, and that makes me a bit relaxed. But it does not mean I have no fear now. I still have it, but we have this professional obligation as health workers, right? So we have to push ourselves. We are like soldiers. Soldiers do not go away when an attack comes, they even go closer and fight. The same is true for us. (Petol, clinical nurse)*

Our participants reported that their fear has reduced over time. Work experience is the main contributor to this change. Moreover, a sense of obligation provided the energy to fight the fear of infection and carry out daily tasks.

### Theme 3: Risk of Contracting DR-TB

There were differences in what the participants reported concerning the existence of risk at their workplace. Interestingly enough, some participants reported that other settings, such as non-TB wards and public transport, are riskier than DR-TB wards and labs for DR-TB infection:

*I think in other wards like OPD (outpatient department), the risk of DR-TB infection is higher since you don’t know if the patients are infected with DR-TB or not. But here, since you know you are working with DR-TB patients, you can try to protect yourself” (Alto, clinical nurse)*

*“For me, the public transport is riskier. We have many DR-TB outpatients who come here almost every day to take their medication. So we take the same taxi with them every day that we come to work. And you know how suffocated and crowded the taxis and buses are, right?”(Pesar, clinical nurse)*

The dominant view here is that DR-TB isolation wards and TB clinics are not the riskiest places in terms of DR-TB infection among HCWs. However, this should not lead to an understanding that these participants consider these places to be non-risky. Nevertheless, these HCWs placed much emphasis on other places, such as non-TB wards and public transport, as being riskier. In non-TB wards, there are patients with both diagnosed and undiagnosed cases. As the HCWs reported, this makes it difficult to know which disease an HCW treats and to take safety measures accordingly. In addition, our respondents expressed concerns over public transport, which they believe is crowded and unventilated. The taxis they mentioned are different from those that operate in other contexts (mainly in the Western world). The taxis referred to here normally carry 12–15 passengers, but they take more people whenever there is less traffic control. Even so, there were also participants who voiced concerns about the risk of contracting DR-TB at the workplace:

*HCWs are at very high risk, and this is why there is no one who wants to work in the TB ward. We are working because we were assigned, and therefore it is a must that we work. (Teman, health officer)*

*Those of us who work in labs are at an especially high risk. I personally know a lab technologist who became an MDR-TB patient. She got infected in the lab. Even though I open windows and ventilate the room, still ….I collect sputum samples, and that means I touch them. So I don’t escape infection. (Kofi, lab technologist)*

### Theme 4: Heavy Workload

The HCWs stated that the available workforce is highly disproportionate to the workload. The amount of work, which includes both handling patient cases and paper-based (reporting) work is high, whereas there is a small number of service providers. As a result, the HCWs have experienced work-related fatigue, with one of them saying: “We have a shortage of manpower. There are times that we spend 24 h without sleep. This is not good” (Ambal, clinical nurse). In addition to sleepless nights, the participants mentioned other consequences, such as an inability to treat patients the way they believe they should. The link between workload and the quality of DR-TB treatment was demonstrated:

*When we work with too many cases per day, then we don’t have the energy to even properly talk to our patients. We become easily irritated by our patients and shout: “Be quick and tell me what you are feeling, otherwise I will call the next patient.” No one understands us. I am working just because I like my profession, despite such challenges. It should not be only about putting bricks together and producing hospital buildings. There should also be a concern to ensure quality services to patients. (Hansir, clinical nurse)*

### Theme 5: Resource Limitations

Participants commented on the availability of resources to carry out their daily tasks. They emphasized that their work environment is poorly equipped, thereby not only constraining them from being effective in their work but also putting them at risk of DR-TB infection. One HCW explained, with a broken voice and eyes filled with tears:

*To your surprise, we are using one mask for 2 weeks or even one per month. And these masks are not the appropriate ones, they are just normal masks. In addition, we asked the health authorities to give us biosafety cabinets, and they said no (biosafety cabinets are an enclosed and ventilated workspace used to work with a contagious material—a sputum sample in the context of this study—in the laboratory). The authorities said that biosafety cabinets are for laboratories at research centres and big hospitals. But this cabinet is very important for us too. (Kofi, laboratory technologist)*

Another participant added that “Our offices and rooms should be comfortable to spend enough time with patients and take a medical history properly” (Melsa, clinical nurse)*.* The predominant perception we found among our participants is that their work environment is characterized by, inter alia, a shortage of resources. The rooms are not comfortable and convenient enough to take the right amount of time with the patients. There is also a shortage of masks and laboratory equipment. The participants voiced concerns about the risks that result from working in such resource-scarce environments.

Reasonable payment was the other lacking resource our participants emphasized. There were different ways in which they explained why they were unhappy with their salary. First, they compared what they earn with what other similar (in terms of how long they studied) professionals earn, and they are dissatisfied when they realize that they are earning much less. One HCW was sad, and took a deep breath after saying this:

*You should not think of your kitchen and your pocket while treating your patients. If you ask me to leave this profession, I will not do it because I love what I am doing. However, it is a disgusting profession, I tell you! You have studied the same number of years as others in other professions but your payment is way too low than what they earn. That is why many health workers these days are studying something else and leaving the profession. (Kash, clinical nurse)*

Second, they refer to the risky nature of their work and feel that they earn much less than what someone who works with such risk should earn. One participant said, “Even though we are at risk here, we do not have a risk allowance. There is no incentive like an additional payment or education opportunity. No one understands our situation. They (health authorities) just say ‘you have an obligation to serve your community’ and do not listen to us. This participant continued, “This does not have that much of an impact on our job. We do our best” (Kotno, clinical nurse). Despite the challenge of earning little, the participants believed that they were doing their best to carry out their daily tasks.

## Discussion

This qualitative study in central Ethiopia explored the challenges of working in DR-TB care in a developing context. Analysing the data from in-depth interviews with 18 HCWs, we found that HCWs experienced the following challenges: 1) inadequate training and provision of information on DR-TB to HCWs assigned to work in DR-TB services, 2) a fear of DR-TB infection, 3) a risk of contracting DR-TB, 4) a heavy workload, and 5) resource limitations. Despite the heterogeneity of participants’ training and the amount of work experience, these experiences were mostly the same across all HCWs. The findings are reviewed below, along with a discussion of their relations to existing knowledge, study limitations, and implications for future work.

Our findings confirm earlier studies by identifying formal education as the least important source of knowledge about DR-TB [35]. We found that HCWs are surprised when they encounter DR-TB cases, which is also reported in other similar developing contexts [36]. Working with patients was the most reported source of knowledge for our participants, followed by colleagues, a few days of TB-specific training (a maximum of 5 days), and self-learning. A total of 44.4% of our participants received on-the-job training on DR-TB, which they perceived as inadequate. This figure is higher than what we found in a previous study conducted among HCWs selected from primary healthcare facilities in Indonesia (29%) [36]. This difference is perhaps because nearly half of our participants (55.5%) were from TB-specialized referral hospitals, where HCWs have a better opportunity to receive such training. In support of this explanation, our figure is in agreement with a Nigerian study (46.9% of its participants received such training), which included participants from a TB referral hospital [37]. Several studies have demonstrated the significant contributions workplace learning and self-education have in improving the knowledge and skills of HCWs [38–40].

Our participants expressed their fear of working with DR-TB patients, a finding that aligns with previous studies, which have shown that HCWs are concerned about being infected with TB [41], and feel stressed and fearful in conducting DR-TB care [36]. In addition to DR-TB infection, their fear resulted from attending to the tragic experiences of DR-TB patients; it resulted from observing DR-TB patients’ experience of going through a long and difficult treatment course. This is in line with a previous study that demonstrated health workers’ fear of DR-TB resulting from, among other things, the disease’s treatment course and drugs [42]. Yet, our participants reported that their fear of infection and frustration grew less the more time they spent working with DR-TB patients. Additionally, a sense of obligation helped them fight their fear [36]. Our findings also showed that although HCWs were fearful of contracting DR-TB, a finding that aligns with several studies on the risk of TB infection among HCWs [10, 26], they acknowledged that there was also a risk of contracting DR-TB in other wards and health facilities where patients may have undiagnosed DR-TB. Moreover, some HCWs perceived more risk in other places such as public transport, due to the fact that it is relatively less difficult to protect oneself at work given DR-TB cases are known and identified. Similar studies show that HCWs consider places other than their work environment as riskier for TB infection [43, 44].

Another key finding is related to the participants’ view of workload and its effect on their ability to carry out daily tasks. The disproportion between the available human resource and the number of patients due to an increase in DR-TB cases negatively affects the quality of treatment services the HCWs provide. The scarcity of HCWs is a serious issue today [45, 46], with high-burden TB countries such as Ethiopia suffering from a shortage in the TB workforce [10].

The participants in our study openly discussed their challenges in facing a high workload, but a low salary. This was found to affect motivation, and bring a lack of interest in the health profession in general. Several studies show that low compensation can negatively affect both health workers’ motivation and their work [26, 47, 48]. As our participants highlighted, resources are generally scarce. This was perceived to affect the health services they provide to patients, insofar as the small and uncomfortable rooms or offices make giving time to patients and taking a detailed medical history difficult, with the lack of laboratories and other equipment constraining a timely diagnosis. Several studies also show that resource limitations at healthcare facilities interrupt the provision of quality treatment to patients [48–52].

The findings of this study are likely to be related to each other within the study’s aim and in accordance with existing literature. For example, and most importantly, it can be interpreted that resource scarcity plays a significant role in increasing the risk and fear of infection, causing a higher workload among healthcare providers. In high-burden TB countries, nearly 80% of TB infections among HCWs are attributed to exposure in healthcare settings, with one of the main contributors to this statistic being inadequate healthcare settings and a shortage of protective equipment [53]. As the evidence shows [54], having inadequate facilities in the workplace increases the risk of contracting DR-TB, and creates infection anxiety among healthcare providers. The same is true for the workload among HCWs, as a lack of medical resources is reported to be one factor for a high nursing workload and burnout [55]. We propose that employing the view that DR-TB is a disease of poverty [11] in the Ethiopian context can further explain the effect of resource limitation on effective DR-TB care.

The present study used a small sample size, thereby making it unlikely to generalize the findings to the perspectives of HCWs in other contexts. Another limitation of this study is that the interviews were conducted during work hours, which might have prevented longer discussions and a deeper investigation of concepts. An additional limitation is that even though most of the participants were recruited by the data collector, some were selected by medical directors themselves. Despite the data collector’s effort to ensure voluntary participation, this might have put pressure on a few of the participants to accept the invitation to participate. Despite these limitations, this study contributes towards a better understanding of the challenges of working in DR-TB care.

To address the increasing burden of DR-TB in resource-limited settings, the challenges and obstacles front-line HCWs have to contend with have to be addressed. Much of the knowledge that our study participants used does not stem from formal education. That is not without its challenges, thus posing the question of how such knowledge can be improved. It is a customary approach to provide knowledge in formal training prior to people starting to work. However, in a situation in which most of our interviewees deem their formal training to be insufficient, it seems more promising to engage with their knowledge in on-the-job training, to expand it, and to amend where necessary. Moreover, given the important job that HCWs deliver, shortages of equipment and remuneration deserve to be addressed. The contribution of this study has also been to inform programmes to acknowledge that HCWs dealing with global pandemics such as TB experience a fear of infection, and identify themselves as a high-risk group. Policymakers should involve grassroots-level HCWs in decision-making so that policies also reflect health providers’ perspectives. The findings of this study warrant a further investigation into healthcare facilities’ service readiness for TB care and applicable modalities to help improve health workers’ motivations.

## Data Availability

The raw data supporting the conclusion of this article will be made available by the authors, without undue reservation.
